# Controversial Aspects Displayed by Enterococci: Probiotics or Pathogens?

**DOI:** 10.1155/2020/9816185

**Published:** 2020-03-20

**Authors:** Moreno Bondi, Andrea Laukova, Simona de Niederhausern, Patrizia Messi, Chrissanthy Papadopoulou, Vangelis Economou

**Affiliations:** ^1^Department of Life Sciences, University of Modena and Reggio Emilia, Via Campi 287, Modena, Italy; ^2^Institute of Animal Physiology, Centre of Biosciences of the Slovak Academy of Sciences, Šoltésovej 4-6, 040 01 Košice, Slovakia; ^3^Microbiology Department, Faculty of Medicine, School of Health Sciences, University of Ioannina, Ioannina, Greece; ^4^Department of Hygiene and Technology of Foods of Animals Origin, Faculty of Veterinary Medicine, School of Health Sciences, Aristotle University of Thessaloniki (AUTH), Greece

*Enterococci* are facultative anaerobic, nonspore-forming Gram-positive bacteria belonging to the Lactic Acid Bacteria (LAB) from the phylum Firmicutes. They are tolerant of a wide range of environmental conditions, surviving in extreme temperature, pH, and sodium chloride concentrations, being found in soil, aquatic environment, plants, sewage, foods, and water, and are one of the standard bacterial indicators for the drinking and recreational water quality [1, 2]. Enterococci are colonizing the gastrointestinal tract of humans and animals (including insects and invertebrates) being part of the gut commensal microbiota. Their name comes from the Greek words “entero” (“*έντερο*”) meaning “intestine” and “coccus” (“*κόκκος*”) meaning “spherical particle,” perfectly describing their origin and morphology together [3].

For centuries, selected enterococcal species have been widely used in the production of a variety of fermented and nonfermented food products ranging from dairy and meat products to vegetable and sea foods [4]. Enterococcal strains can produce bacteriocins, some of which are heat-stable peptides with low molecular weight exhibiting remarkable antibacterial activities. Enterococci also have properties that are of technological interest in the food industry, and some strains have been used as probiotics for the maintenance of normal intestinal microbiota, stimulation of the immune system, and improvement of the nutritional value of foods and feeds in humans and animals [5, 6].

However, following the emergence of antibiotic-resistant (AMR) enterococci and particularly of the vancomycin-resistant enterococci (VRE), these microorganisms have turned from generally recognized as safe (GRAS) for human consumption to significant pathogens threatening human health and thriving in the hospital environment. According to World Health Organization (WHO), VREs are pathogens of high priority in the list of microorganisms for which the development of new antimicrobials is an urgent demand [7]. Two species (*E. faecalis* and *E. faecium*) which are widely used as probiotics have been implicated in severe infections of the central nervous system, urinary tract, intra-abdominal and pelvic infections, endocarditis, and bacteremia [8]. Enterococci display important biological traits including the presence of drug-resistant genes, the production of cytolysin, adhesins, invasins, and gelatinase, which contribute to their virulence and ability to colonize tissues [9, 10].

Thus, recently the trend of using enterococci as probiotics for human consumption is in debate due to the controversial aspects of these bacteria which appear to be “friends and foes” [11]. There are published studies reporting that GRAS probiotics are causing infections, but there are no published reports that enterococcal probiotics cause human infections. Hence, taking into consideration the diversity of strains within each bacterial species and the impressive potential of the microorganisms to reorganize their genomes in their eternal effort for survival, the question whether enterococci are beneficial probiotics or dangerous pathogens is very intriguing and may take long to be answered justifiably.

This special issue contains six articles reporting enterococci isolation from foods and sewage sludge, the presence of virulence genes, the antimicrobial resistance of *E. faecalis* and *E. faecium* isolates from humans and food, and the safety aspects and probiotic properties of *E. faecium* FL31 producing enterocin BacFL31, as well as a review summarizing the pros and cons of enterococci as probiotics and emerging pathogens.

The study by Maasjost et al. reports the presence of virulence genes detected in enterococcal species isolated from meat of turkeys. The isolates belonged to three species (*E. faecalis*, *E. faecium*, and *E. gallinarum*) and were examined for common virulence genes and their phenotypic expression. All isolates were analyzed for five selected putative virulence traits to explore their potential role in the pathogenicity using the chicken embryo lethality assay. The results differ markedly between the three *Enterococcus* species, with *E. faecalis* harboring the majority of the investigated genes and virulence traits. From the results of this study, it is clear that the presence or absence of virulence genes or corresponding phenotypes does not entirely correlate with the isolates' virulence potential and pathogenicity for chicken embryos.

Golob et al. in their study determine and compare the antimicrobial susceptibility and virulence traits of *E. faecalis* and *E. faecium* isolates from human clinical specimens and retail meat (fresh beef and pork). All isolates were investigated for susceptibility to 12 antimicrobials using a broth microdilution method and for the presence of seven common virulence genes using PCR. The results are quite favorable as all isolates were susceptible to daptomycin, linezolid, teicoplanin, and vancomycin with a considerably higher proportion of susceptible isolates from meat compared to clinical isolates (only 1.7% of meat isolates were multidrug resistant compared to 42.6% of the clinical isolates). The findings of this study show that *E. faecalis* and *E. faecium* from red meat most likely do not represent an important source of resistant strains to human consumers.

Laukova et al. report the isolation of four different enterococcal species from trouts in Slovakian water sources. The four species (*E. durans*, *E. faecium*, *E. mundtii*, and *E. thailandicus*) were identified using matrix-assisted laser desorption/ionization time-of-flight mass spectrometry (MALDI-TOF-MS). The hemolytic, gelatinase, and nuclease activity determined by cultural techniques was found negative, while the enzymatic activity tested by biochemical and spectrophotometric methods was acceptable. All strains possessed gene for enterocin A production, and all strains were susceptible to antibiotics which is a very positive finding. This study reports in detail the properties of enterococci isolated from trout shedding more light into species isolated from wild sources.

Another study by Laukova et al. concerns the incidence of virulence factor genes among enterococci isolated from sewage sludge (cow's dung water). Species identification of 24 enterococcal strains by ΜALDI-TOF-MS allotted 23 strains to the species *E. faecium* with highly probable species identification and *E. faecalis* EEV20 with a score value meaning secure genus identification/probable species identification. Enterococci were absent of *cytolysin A* gene, *hyaluronidase* gene, and *element IS* gene. It is concluded that they were not invasive which is very important from the safety side. According to the results of this study, the most frequently detected gene was adhesin *E. faecium* (*efaAfm*, in 22 *E. faecium* strains and in one *E. faecalis*).

The safety aspects and probiotic properties of *E. faecium* FL31 strain producing enterocin BacFL31 in combination with the aqueous peel onion (*Allium cepa*) extract (APOE) in ground beef meat storage are explored in the study by Mtibaa et al. The biopreservative effect of two natural compounds (bacteriocin BacFL31 and APOE) added alone or in combination was evaluated by microbiological, physicochemical, and sensory analyses during 14 days at 4°C. The results show that the combination of APOE and BacFL31 was significantly more effective than the use of each active compound alone, limiting the microbial deterioration, decreasing thiobarbituric acid-reactive substances, slowing down metmyoglobin (MetMb) and carbonyl group accumulation, delaying the disappearance of sulfhydryl proteins, inhibiting efficiently the microflora proliferation, and indicating that enterocin BacFL31 derived from a safe *Enterococcus faecium* and combined with APOE is a promising natural preservative for ground beef.

Braїek and Smaoui review the pros and cons of enterococci in view of their future use as probiotics and discuss their dual and controversial features between opportunistic pathogens and promising probiotics providing a useful overview of the existing knowledge on their taxonomy, physiological and biochemical traits, habitats, occurrence in different foods, enterocin classification, spectrum and mode of action, pathogenicity, virulence factors, antimicrobial resistance (AR), transfer of virulence factors, and AR genes and finally discuss enterococci as probiotics.

We would like to thank all the authors for their contributions in this special issue and acknowledge all the reviewers for their time spent in assessing the submitted manuscripts. Also, we thank the editorial office of the *BioMed Research* journal for their assistance throughout the completion of this special issue.

*Moreno Bondi* *Andrea Laukova* *Simona de Niederhausern* *Patrizia Messi* *Chrissanthy Papadopoulou* *Vangelis Economou*
